# Supplementary material for: Impact of Extreme Temperatures on Ambulance Dispatches Due to Cardiovascular Causes in North-West Spain
Source: Int J Environ Res Public Health. 2020 Dec 3;17(23):9001. doi: 10.3390/ijerph17239001 (PMC7729967; doi:10.3390/ijerph17239001)
Supplement: Supplementary file 1 [file ijerph-17-09001-s001.pdf]

Supplementary

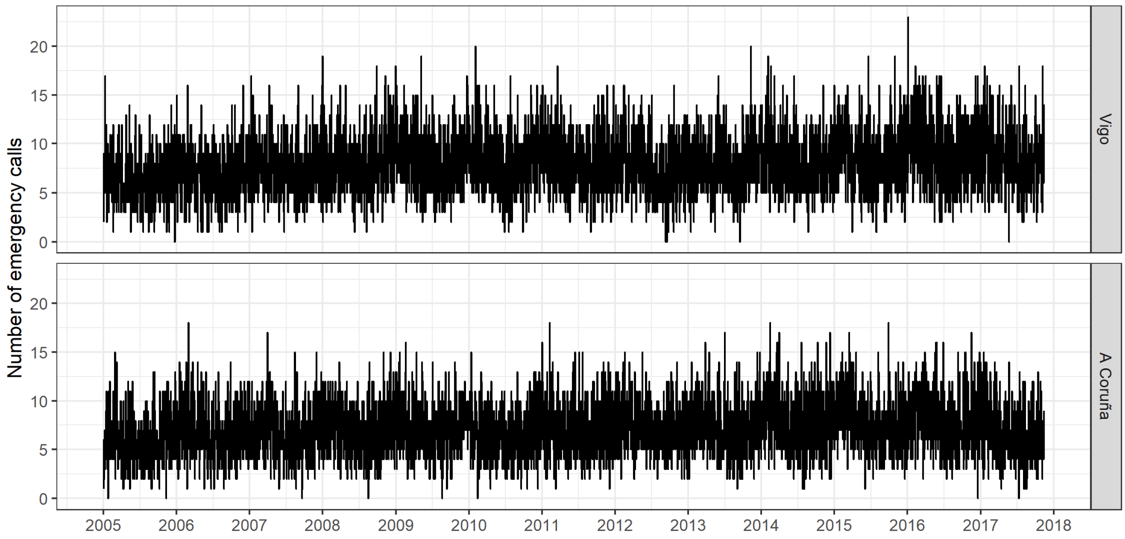

Figure S1. Daily series of emergency medical calls due to cardiovascular causes in Vigo and A Coruña from 2005 to 2017.

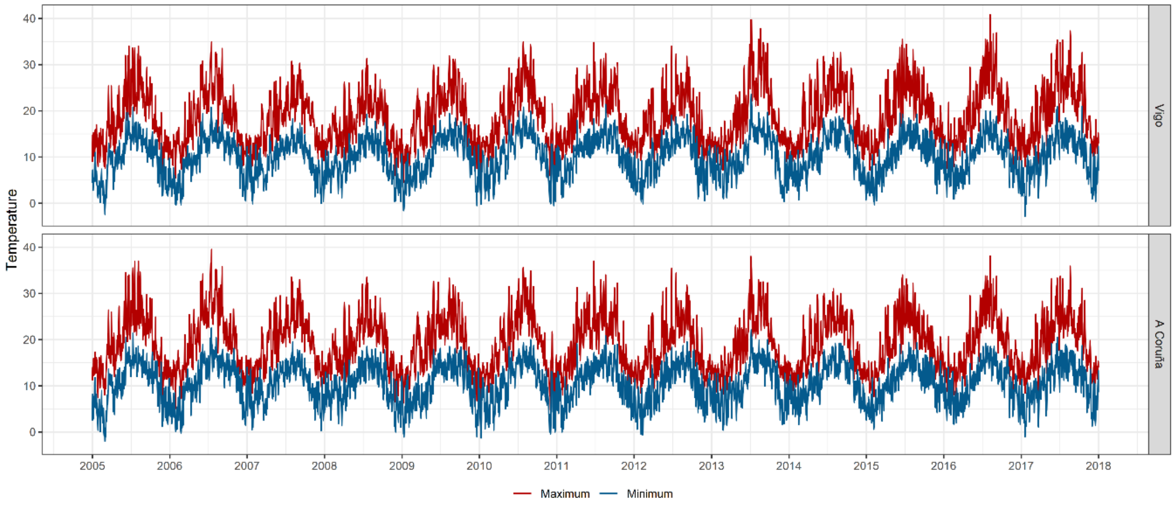

Figure S2. Daily series of Tmax and Tmin in Vigo and A Coruña.
